# Correction: Sun et al. Ergosterol Isolated from *Antrodia camphorata* Suppresses LPS-Induced Neuroinflammatory Responses in Microglia Cells and ICR Mice. *Molecules* 2023, *28*, 2406

**DOI:** 10.3390/molecules28217236

**Published:** 2023-10-24

**Authors:** Ping Sun, Weiling Li, Jiazheng Guo, Qian Peng, Xiansheng Ye, Song Hu, Yuchen Liu, Wei Liu, Haifeng Chen, Jialu Qiao, Binlian Sun

**Affiliations:** 1 Wuhan Institute of Biomedical Sciences, School of Medicine, Jianghan University, Wuhan 430056, China; 2 Fujian Provincial Key Laboratory of Innovative Drug Target, School of Pharmaceutical Sciences, Xiamen University, Xiamen 361005, China

## Error in Figure

The original version of this article unfortunately contains an error in Figures 4B,C,H–J and 5C,D [1]. The unit of LPS and Ergosterol in histogram is (mg/kg). The correct version of Figure 4B,C,H–J is given below.

The correct version of Figure 5C,D is given below.

The authors apologize for any inconvenience caused and state that the scientific conclusions are unaffected. This correction was approved by the Academic Editor. The original publication has also been updated.

## Figures and Tables

**Figure 4:**
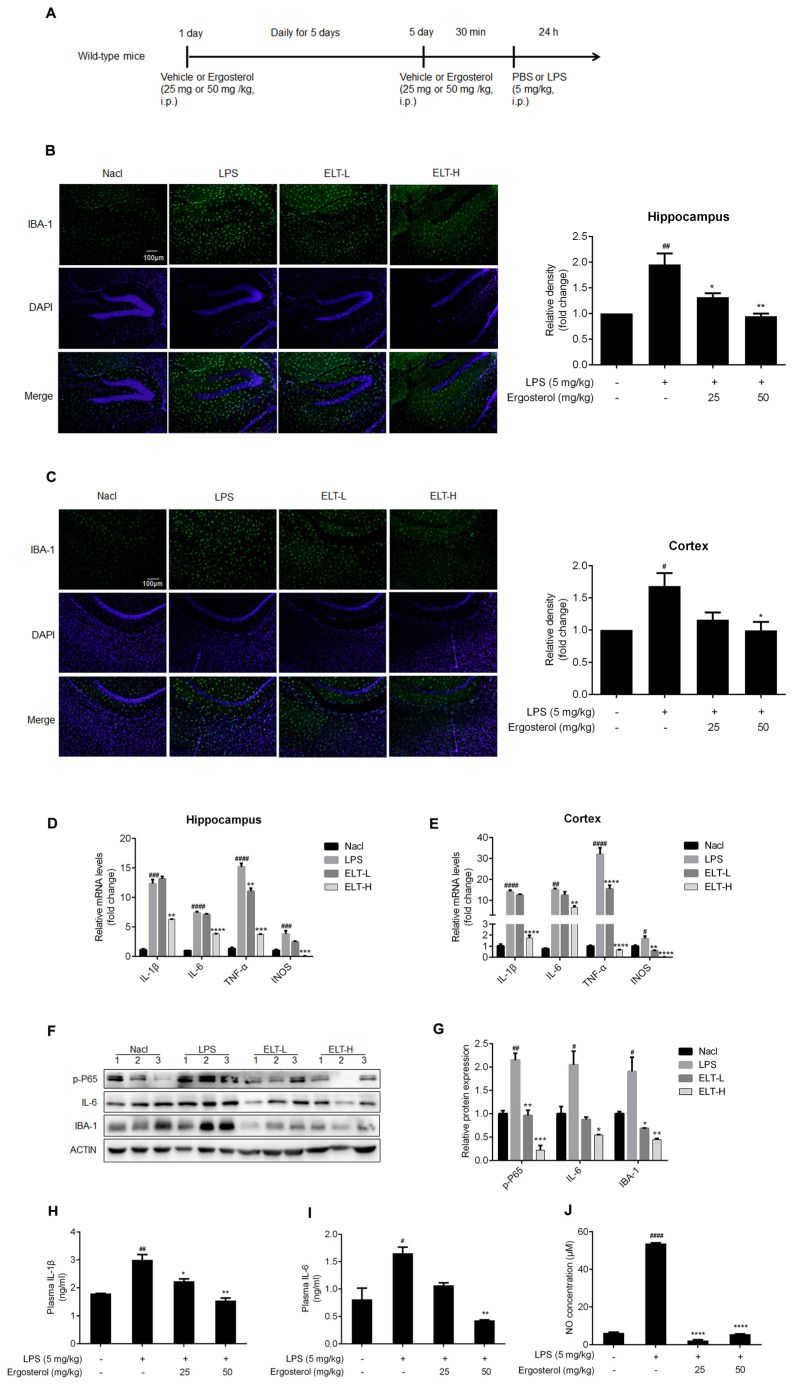
Ergosterol markedly decreased LPS-stimulated microglial activation and inflammation in ICR mice. (**A**) ICR mice were injected daily with Ergosterol (25 mg/kg, 50 mg/kg, i.p.) or vehicle (PBS, i.p.) for 5 days, followed by LPS (5 mg/kg, i.p.) or PBS injected for 24 h. The mice were then perfused and fixed. (**B**,**C**) An anti-IBA-1 antibody was used for immunohistochemistry. Quantification of IBA-1 fluorescence strength in the cerebral cortex or hippocampus (Nacl, *n* = 8 mice; LPS, *n* = 8 mice; Ergosterol 25 mg/kg + LPS is marked as ELT-L, *n* = 8 mice; Ergosterol 50 mg/kg + LPS is marked as ELT-H, *n* = 8 mice). (**D**,**E**) The mRNA levels of the IL-1β, IL-6, TNF-α, and INOS inflammatory factors in the cortex and hippocampus of the mice were measured with qRT-PCR. (**F**) The p-P65, IL-6, and IBA-1 protein levels were determined by Western blot. Actin was used as a housekeeping control (Nacl, *n* = 3 mice; LPS, *n* = 3 mice; ELT-L, *n* = 3 mice; ELT-H, *n* = 3 mice). (**G**) The relative expression level of protein to actin was calculated by densitometry. (**H**,**I**) The protein levels of IL-1β and IL-6 in plasma were detected by ELISA. (**J**) The expression levels of NO in plasma were detected with a Griess assay. Statistical significance was analyzed using one-way ANOVA and *t* test. # Significantly different from the control; * significantly different from the LPS-treated group. Significance: ^#^ *p* < 0.05, ^##^ *p* < 0.01, ^###^ *p* < 0.001, ^####^ *p* < 0.0001, * *p* < 0.05, ** *p* < 0.01, *** *p* < 0.001, and **** *p* < 0.0001.

**Figure 5:**
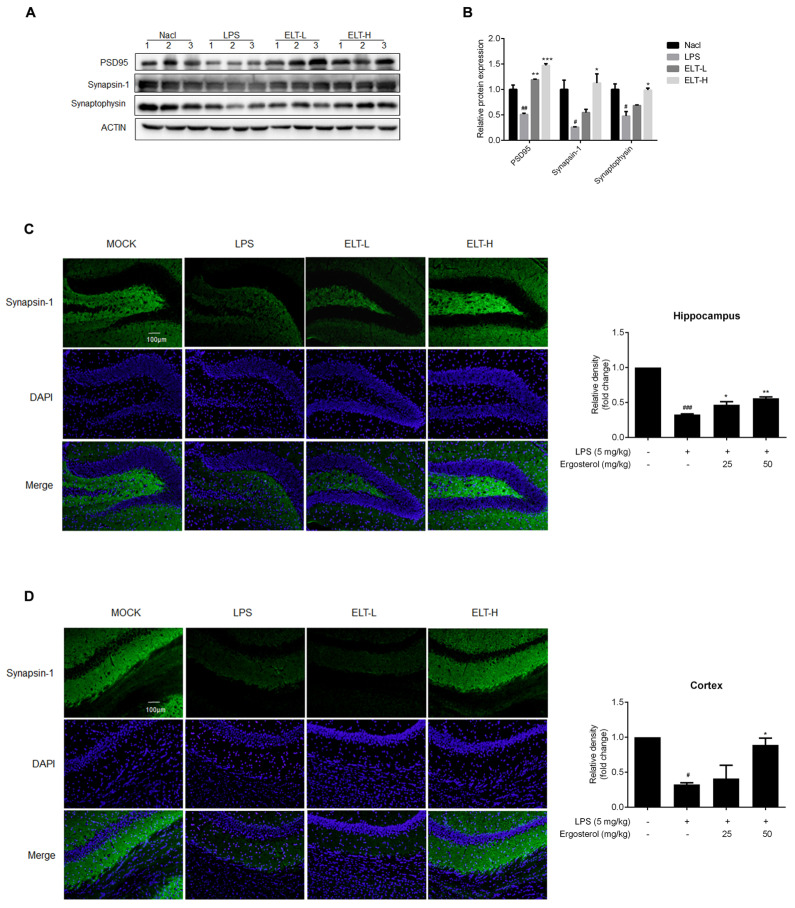
Ergosterol significantly reduced LPS-induced neuron injury in ICR mice. (**A**) The PSD95, Synapsin-1, and Synaptophysin protein levels in the cortex of mice were determined by Western blot. Actin was used as a housekeeping control (Nacl, *n* = 3 mice; LPS, *n* = 3 mice; ELT-L, *n* = 3 mice; ELT-H, *n* = 3 mice). (**B**) The densitometric method was used to estimate the relative expression of the protein compared to actin. (**C**,**D**) Immunohistochemistry was performed on anti-Synapsin-1 antibody. DAPI was used as a marker of the nucleus. Quantification of the relative fluorescence strength of Synapsin-1 in the region of the cortex or hippocampus was analyzed (Nacl, *n* = 8 mice; LPS, *n* = 8 mice; ELT-L, *n* = 8 mice; ELT-H, *n* = 8 mice). All data are mean ± SEM of triplicate values. Statistical significance was analyzed using one-way ANOVA and *t* test. # Significantly different from the control; * significantly different from the LPS-treated group. Significance: ^#^ *p* < 0.05, ^##^ *p* < 0.01, ^###^ *p* < 0.001, * *p* < 0.05, ** *p* < 0.01, and *** *p* < 0.001.
