# Supplementary material for: Ergosterol Isolated from Antrodia camphorata Suppresses LPS-Induced Neuroinflammatory Responses in Microglia Cells and ICR Mice
Source: Molecules. 2023 Mar 6;28(5):2406. doi: 10.3390/molecules28052406 (PMC10005213; doi:10.3390/molecules28052406)
Supplement: Supplementary file 1 [file molecules-28-02406-s001.zip › molecules-2211712-supplementary.pdf]

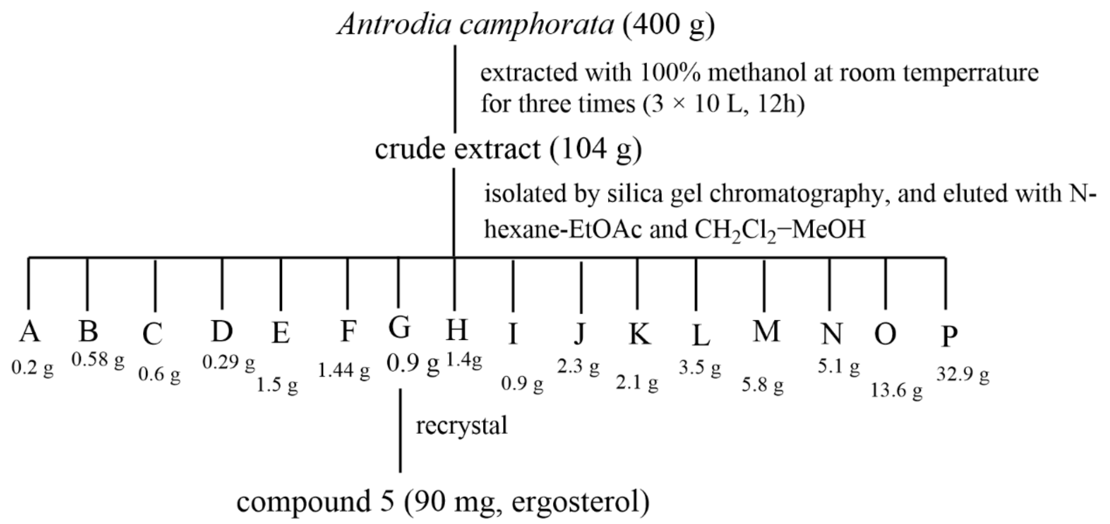

Figure S1. The separation process of compound **5** (Ergosterol).

Table S1. ^{1}H NMR and ^{13}C NMR Spectroscopic Data for Compound 5 (*δ* in ppm, *J* in Hz) in $CDCl_3$

position	δ_H	δ_C
1	2.4 (1H, m); 1.21 (1H, m)	38.4
2	1.98 (1H, m); 1.97 (1H, m)	32.0
3	3.57 (1H, tt, *J* = 11.1, 4.1 Hz)	70.5
4	1.85 (1H, m); 1.83 (1H, m)	40.8
5		139.8
6	5.50 (1H, dd, *J* = 5.5, 2.2 Hz)	119.6
7	5.32, (1H, m)	116.3
8		141.4
9	1.82 (1H, m)	46.2
10		37.0
11	1.62 (1H, m); 1.21 (1H, m)	21.1
12	1.57 (1H, m); 1.81 (1H, m)	39.1
13		42.8
14	2.22, (1H, m)	54.6
15	2.00 (1H, m); 1.41 (1H, m)	23.0
16	1.81 (1H, m); 1.30 (1H, m)	28.3
17	1.21, (1H, m)	55.7

18	0.56, (3H, s)	12.1
19	0.88, (3H, s)	16.3
20	1.23, (1H, m)	40.4
21	0.97, (3H, d, *J* = 6.8 Hz)	21.1
22	5.10 (1H, dd, *J* = 15.3, 7.3 Hz)	135.6
23	5.16 (1H, dd, *J* = 15.3, 7.3 Hz)	132.0
24	1.41, (1H, m)	42.8
25	1.43, (1H, m)	33.1
26	0.76 (3H, d, *J* = 6.8 Hz)	20.0
27	0.77 (3H, d, *J* = 6.8 Hz)	19.7
28	0.85 (3H, d, *J* = 6.8 Hz)	17.6

^{1}H NMR spectra measured at 600 MHz, ^{13}C NMR spectra measured at 150 MHz.

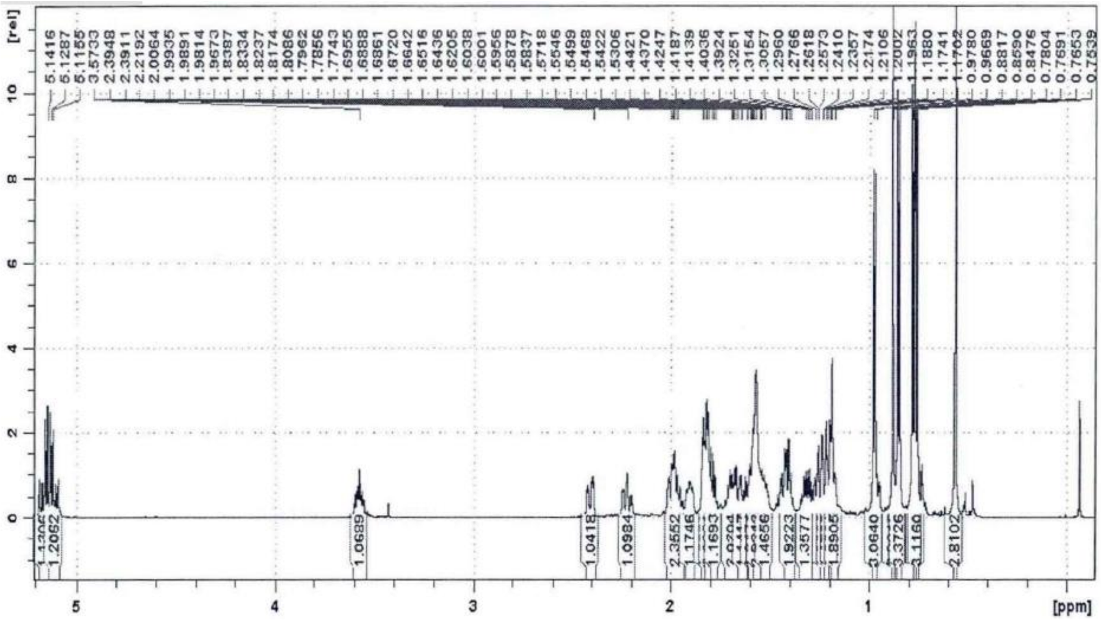

Figure S2. The ^{1}H-NMR spectrum of compound **5** (Ergosterol).

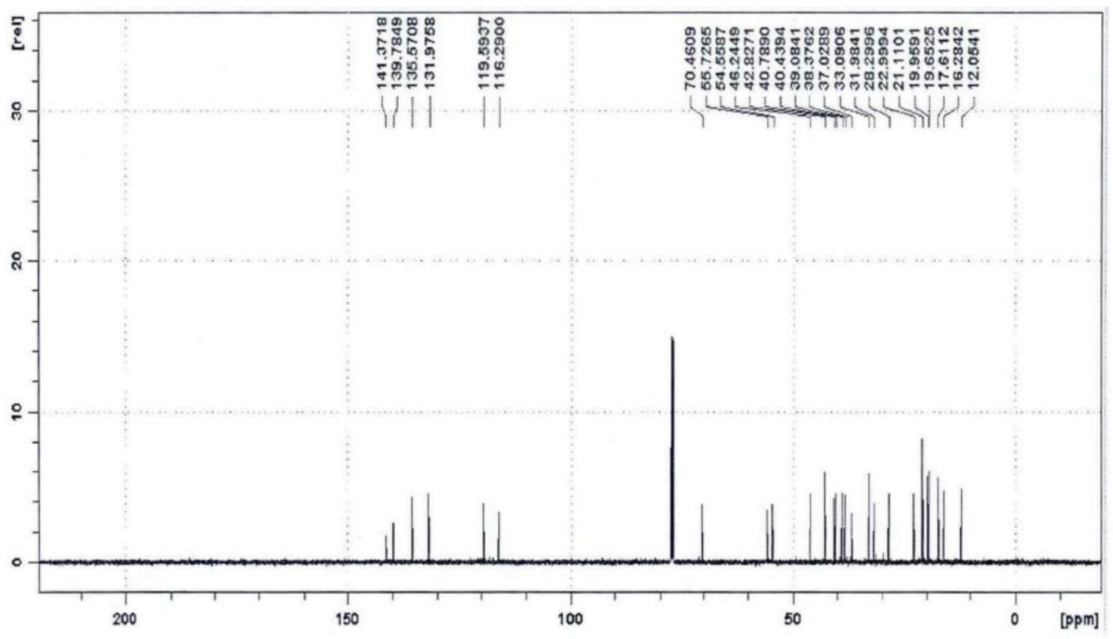

Figure S3. The ^{13}C-NMR spectrum of compound **5** (Ergosterol).

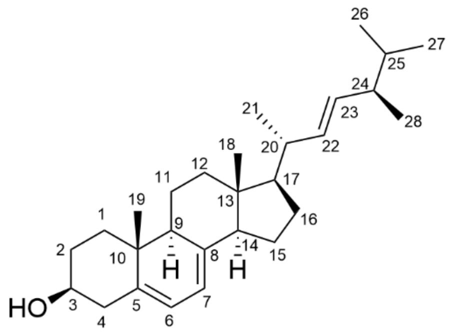

Figure S4. The structure of compound **5** (Ergosterol).

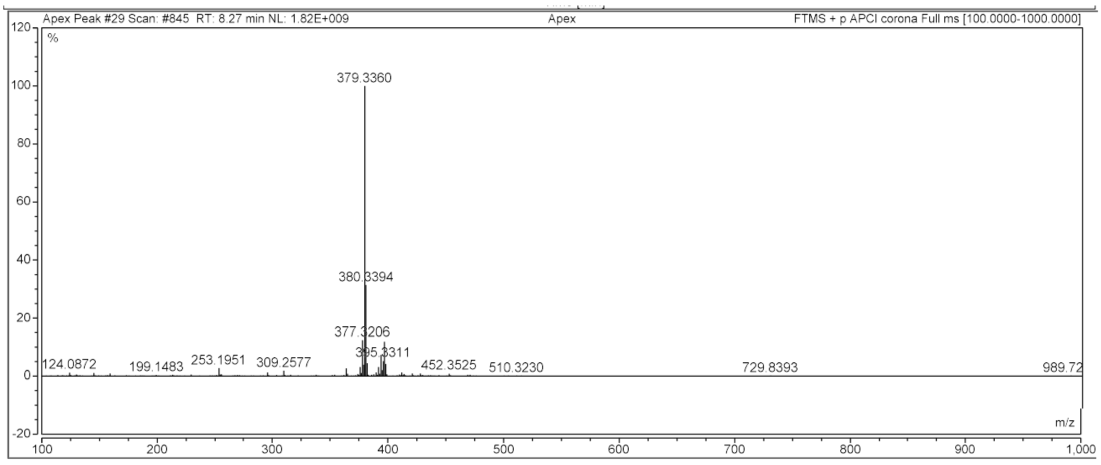

Figure S5. The HRESIMS spectrum of compound **5** (Ergosterol).

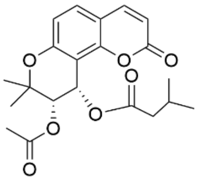

corymbocoumarin

Figure S6. The structure of compound **24** (corymbocoumarin).

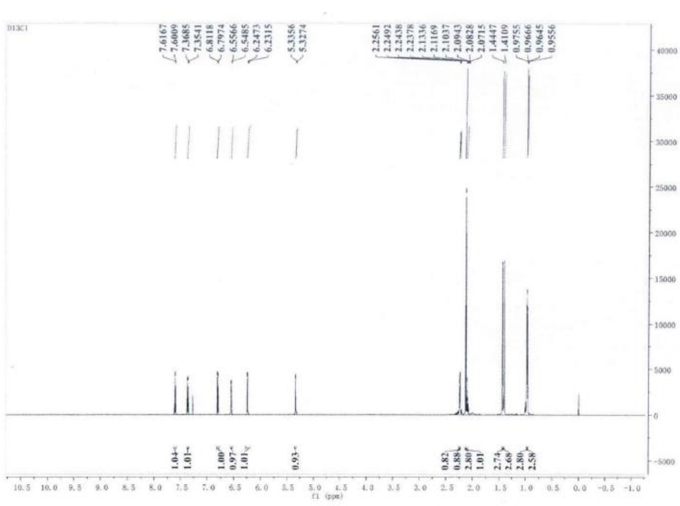

Figure S7. The ^{1}H-NMR spectrum of compound **24.**

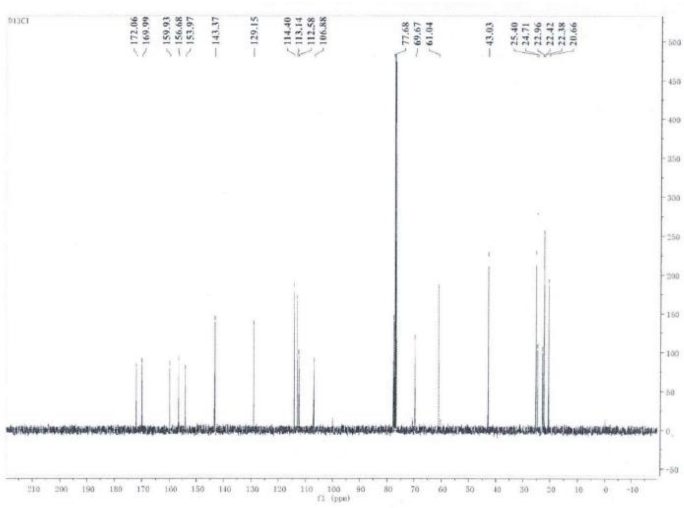

Figure S8. The ^{13}C-NMR spectrum of compound **24**.

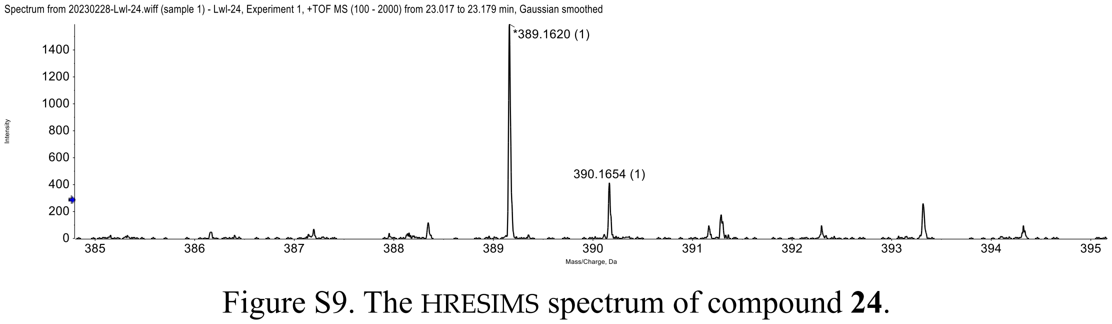

Figure S9. The HRESIMS spectrum of compound **24**.

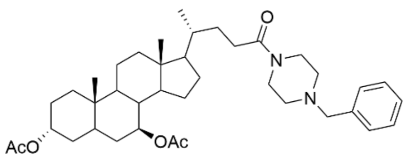

Ua1

Figure S10. The structure of compound **48** (Ua1).

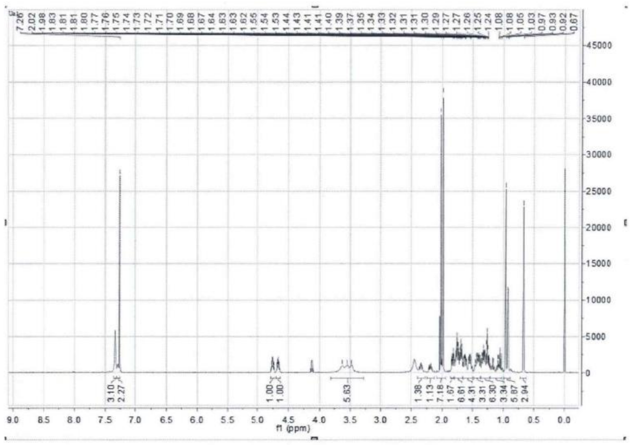

Figure S11. The ^{1}H-NMR spectrum of compound **48**.

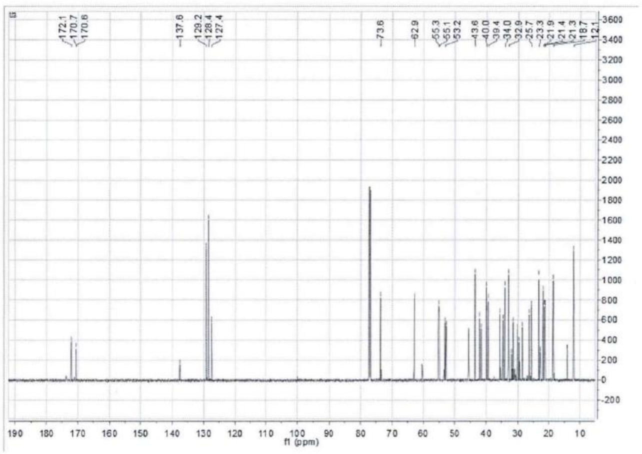

Figure S12. The ^{13}C-NMR spectrum of compound **48**.

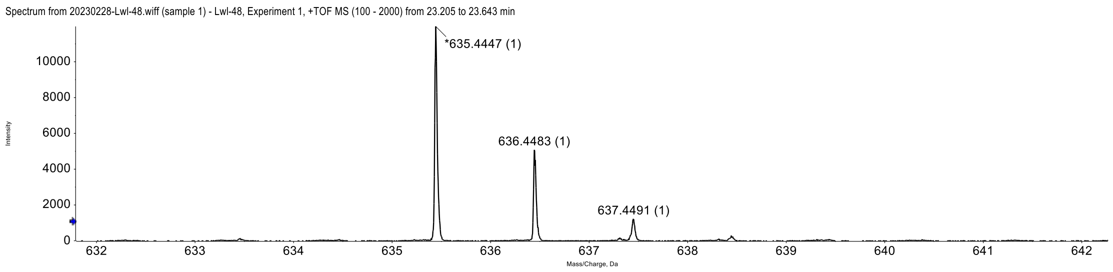

Figure S13. The HRESIMS spectrum of compound **48.**

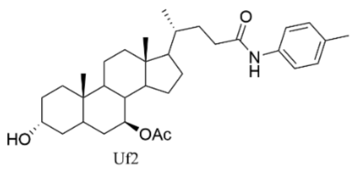

Uf2

Figure S14. The structure of compound **49**.

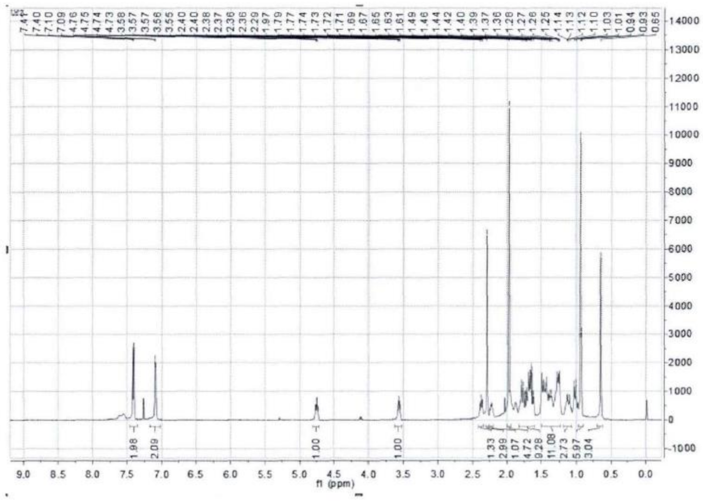

Figure S15. The ^{1}H-NMR spectrum of compound **49**.

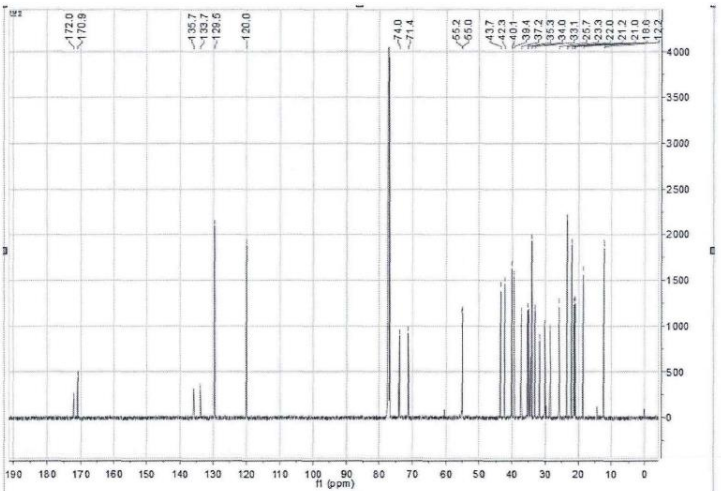

Figure S16. The ^{13}C-NMR spectrum of compound **49**.

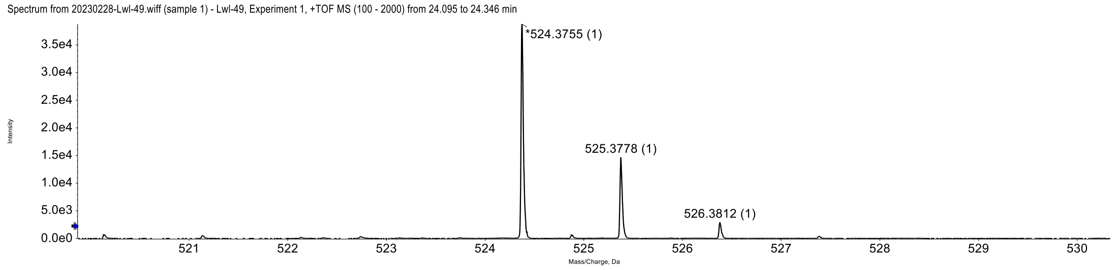

Figure S17. The HRESIMS spectrum of compound **49.**
